# Doctor AI? A pilot study examining responses of artificial intelligence to common questions asked by geriatric patients

**DOI:** 10.3389/frai.2024.1438012

**Published:** 2024-07-25

**Authors:** Ian Moore, Christopher Magnante, Ellie Embry, Jennifer Mathis, Scott Mooney, Shereen Haj-Hassan, Maria Cottingham, Prasad R. Padala

**Affiliations:** ^1^Geriatric Research Education and Clinical Center (GRECC), Central Arkansas Veterans Healthcare System (CAVHS), Little Rock, AR, United States; ^2^Tennessee Valley Veteran Affairs Healthcare System (TVHS), Nashville, TN, United States; ^3^Department of Psychiatry, University of Arkansas for Medical Sciences (UAMS), Little Rock, AR, United States; ^4^Baptist Health-UAMS Graduate Medical Education, Little Rock, AR, United States

**Keywords:** artificial intelligence (AI), immersive technologies, geriatrics, dementia, patient feedback, diagnosis and treatment planning

## Abstract

**Introduction:**

AI technologies have the potential to transform patient care. AI has been used to aid in differential diagnosis and treatment planning for psychiatric disorders, administer therapeutic protocols, assist with interpretation of cognitive testing, and patient treatment planning. Despite advancements, AI has notable limitations and remains understudied and further research on its strengths and limitations in patient care is required. This study explored the responses of AI (Chat-GPT 3.5) and trained clinicians to commonly asked patient questions.

**Methods:**

Three clinicians and AI provided responses to five dementia/geriatric healthcare-related questions. Responses were analyzed by a fourth, blinded clinician for clarity, accuracy, relevance, depth, and ease of understanding and to determine which response was AI generated.

**Results:**

AI responses were rated highest in ease of understanding and depth across all responses and tied for first for clarity, accuracy, and relevance. The rating for AI generated responses was 4.6/5 (SD = 0.26); the clinician s' responses were 4.3 (SD = 0.67), 4.2 (SD = 0.52), and 3.9 (SD = 0.59), respectively. The AI generated answers were identified in 4/5 instances.

**Conclusions:**

AI responses were rated more highly and consistently on each question individually and overall than clinician answers demonstrating that AI could produce good responses to potential patient questions. However, AI responses were easily distinguishable from those of clinicians. Although AI has the potential to positively impact healthcare, concerns are raised regarding difficulties discerning AI from human generated material, the increased potential for proliferation of misinformation, data security concerns, and more.

## Introduction

Before the term artificial intelligence (AI) was first coined in 1956 (Russell and Norvig, 2016), Alan M. Turing conceived of his famous “Turing test.” With his test, Turing sought to explore whether computer-generated responses could be distinguished from those of humans by unaware observers (Kleppen, 2023). A computer “passes” the Turing test if its responses are indistinguishable from those of real human respondents. In 2014, a chatbot known as Eugene Goostman (Warwick and Shah, 2015), was the first machine to pass the Turing test, representing a significant milestone for AI and machine learning and setting a benchmark for subsequent programs to meet and surpass.

As currently defined, AI refers to computer systems designed to perform tasks that would otherwise require human intervention (Sutton and Barto, 2018). Early AI research focused on general problem solving using symbolic logic and rules-based systems (Jordan and Mitchell, 2015). Initially, AI research was met with optimism (Russell and Norvig, 2016); however, due to inadequate funding and computing power, among other factors, research efforts stagnated. In the 1990's and 2000's, ML advanced significantly due to the advent of neural networks, reinforcement learning, computer vision, and natural language processing (Jordan and Mitchell, 2015) along with the rise of big data, cheaper computing, and advanced computational algorithms. Most recently, deep learning AI models, which are layered networks of machine learning algorithms that can extract increasingly complex information via processing of mass amounts of data, have led to major breakthroughs in AI based research (LeCun et al., 2015). There remains spirited debate about whether an AI has passed the Turing test. Today, in both text and speech, there are numerous claims regarding how deep learning programs (e.g., Chat GPT) and text to speech programs can now pass the Turing test by producing outputs that are indistinguishable from humans (Biever, 2023; Mai et al., 2023).

AI technologies have been particularly transformative to healthcare systems in recent years (Yu et al., 2018). For instance, in medical imaging, deep learning algorithms have been used to detect potential abnormalities with greater accuracy than radiologists (Liu et al., 2019). Natural language processing has enabled AI to analyze and extract relevant health data from patient medical records to assist with accurate diagnosis and aid in treatment planning (Kreimeyer et al., 2017). Wearable AI assisted monitoring systems have been used to track important patient health metrics and can alert caregivers about potential health risks (Senders et al., 2018). AI has also been used in robot-assisted surgery to automate routine tasks and improve precision (Hashimoto et al., 2018). In the pharmaceutical industry, deep learning has been useful in drug development and can even be used to help providers identify medications that would be most effective for patients based on their biological/genetic characteristics and individual needs (Mak et al., 2023). In clinical practice chatbots and virtual assistants have proven beneficial for patient education, medication reminders, and mental health support (Miner et al., 2016).

In mental healthcare, the applications of AI technologies have been similarly impactful. Specifically, AI and ML tools have shown promise for improving the diagnosis and treatment of psychiatric disorders. For instance, natural language processing algorithms have been used to analyze speech patterns and textual data from patients to identify early signs of anxiety, depression, and suicide risk (Inkster et al., 2018) while ML models have successfully been used to develop individualized patient treatment plans. Therapeutically, chatbots and conversational agents have demonstrated emotion recognition capabilities and have used validated therapeutic techniques to provide basic mental health support services (Inkster et al., 2018). In neurology and neuropsychology, AI algorithms have proven beneficial in early diagnosis of neurodegenerative disorders via interpretation of neuroimaging (Sarraf and Tofighi, 2016), while ML methods have been used to assist with scoring and interpretation of neurocognitive testing. AI-based systems have also been used to create personalized cognitive rehabilitation and treatment plans for patients recovering from traumatic brain injuries or strokes (Iaccarino et al., 2018).

Despite these notable advancements, there remains many shortcomings in the use of AI in healthcare settings, especially in dementia and geriatric healthcare. AI has not yet proven itself able to have dynamic, empathetic exchanges with patients who utilize it. Further, patient perceptions of AI involvement in clinical care are generally poor, with research indicating that patients are accepting of AI use by trained providers to assist with their care, but are hesitant to support or desire fully AI driven care personally (Russell and Norvig, 2016). Lastly, and perhaps most concerningly, research has shown that, despite notable pitfalls, lay persons may be overly trusting of AI generated information, which could lead to the proliferation of misinformation in healthcare (Young et al., 2021). Although certainly impressive in their capabilities to synthesize and learn from large amounts of information, AI technologies are not infallible and still require oversight from trained professionals to ensure the accuracy and applicability of the information they generate.

Although AI holds immense potential and promise for revolutionizing patient care, many of the technologies are in their infancy and require further research to determine their utility and applicability in various healthcare settings. In this vein, little research has explored the capabilities and utility of generative AI to the field of dementia and geriatric healthcare. To further understand the strengths and limitations of AI as it relates these fields, this pilot study seeks to do the following:

- Compare AI generated responses to those of trained clinicians with regard to providing patient feedback to commonly asked queries.
- This study will compare the accuracy, relevance, and utility of AI generated feedback to responses generated by trained clinicians.
- Our goal of this study is to highlight potential strengths and limitations of current AI technologies as they relate to answering patient questions compared to trained clinicians and discuss potential future directions for this research.

## Methods

### Participants

Questions used in the study were created by trained clinicians who provided the most commonly asked questions they received during their work with patients and their families. Responses to the finalized questions were solicited from three clinicians who were instructed to respond to the questions as if they were answering them for patients and/or their family members. Chat-GPT 3.5 was the AI program used in this study. Finally, clinician and AI generated responses were sent to a fourth, blinded clinician who rated all responses to each question and attempted to identify AI generated responses. All clinicians in this study are clinical neuropsychologists, with three of four having their board certification in clinical neuropsychology. Importantly, each clinician only participated in one aspect of the study, so those who created the questions did not respond to or rate them. As experts on neurocognitive disorders and geriatric cognition, clinical neuropsychologists represent the “gold standard” against which to compare AI's current capabilities to answer common patient inquiries, which is why these clinicians were chosen for this study.

### Procedures/data collection

#### Clinical question creation

Two clinicians were asked to provide three to five of the most commonly asked questions they received from patients or family members in their clinics. A total of eight potential questions were provided; the two clinicians then voted on the best five questions to include in this study. Importantly, both open ended and closed ended questions were included to test the true capabilities of AI.

Once finalized, the questions were sent to three clinicians who were asked to answer each query as if they were responding directly to patients or their family members. No other restrictions were placed on the clinicians' responses so they could answer them authentically. The questions were then inputted into Chat-GPT 3.5. To ensure that AI generated responses were not identifiable by length, AI was instructed to answer each question in the average number of words of the clinicians' response; but this was the only restriction placed on the AI software. AI and clinician responses to each question were then randomized before being sent to the rater to reduce easy identification and potential rater bias.

As the purpose of this study was to examine responses to questions commonly posed by patients, no training was done with AI to improve its responses and answers were only generated one time. Because this study focuses on questions commonly asked by older adults, we attempted to use AI similarly to how an older adult might. As older adults typically possess low technological literacy than their younger counterparts, it is reasonable to assume that few would understand how to train AI models to generate better responses (Schreurs et al., 2017). As such, all questions were only inputted once and without refinement, as we strongly feel this is how older patients, untrained in AI technologies, would use it to answer their questions when asked to do so.

### Response ratings

Clinician and AI generated responses were sent to a separate clinician for rating. The rater was asked a series of five questions designed to assess the quality of the responses provided by the clinicians and AI to the previously created queries. Specifically, using a five-point Likert scale (1 = poor; 5 = excellent), responses to each question were rated on their clarity, factual accuracy, relevance, depth, and ease of understanding (Appendix Table 2). Additionally, the rating clinician was asked to choose which response they believed was AI generated and provide justification for their answer (Appendix Table 1). Complete responses to all questions by AI and the clinicians are presented in Appendix Table 3.

### Statistical analyses

Means and standard deviations were calculated for each of the clinician and AI generated responses to each question to identify which response(s) was (were) rated the most highly based upon clarity, factual accuracy, relevance, depth, and ease of understanding. Additionally, overall means and standard deviations for clarity, factual accuracy, relevance, depth, and ease of understanding were calculated to determine how each of the respondents performed with regard to each of these criteria. Further, an overall average was calculated to see which respondent's answers were rated most highly overall. Finally, the rater's choice for an AI generated response and their rationale for their choice were presented to see if AI generated responses could be differentiated from those of trained clinicians. Due to the small sample size of questions, respondents and raters used in this study, “gold standard” statistical analysis procedures (Versi, 1992) for evaluating the quality of AI responses compared to trained clinicians were not implemented.

## Results

All questions used in this study and the associated ratings for each of the clinicians and AI generated responses are presented in Tables 1–5. The average of all responses for each clinician and the AI are presented in Table 6. Finally, the rating clinician's choices and justification for identifying the AI generated response to each prompt are presented in Appendix Table 1.

**Table 1:** Question 1 ratings.

**Question 1**	**I think my loved one has dementia. What should my next steps be and what are potential treatment considerations?**			
	**Clinician 1**	**Clinician 2**	**Clinician 3**	**AI**
Clarity	4	5	5	4
Accuracy	5	4	5	4
Relevance	5	4	5	4
Depth	5	2	2	4
Ease of understanding	3	4	5	5
Average (SD)	4.4 (0.89)	3.8 (1.10)	4.4 (1.34)	4.2 (0.45)

**Table 2:** Question 2 ratings.

**Question 2**	**Will taking Prevagen improve my memory? Is it worth the cost?**			
	**Clinician 1**	**Clinician 2**	**Clinician 3**	**AI**
Clarity	5	5	4	5
Accuracy	5	5	4	5
Relevance	5	5	4	5
Depth	4	4	3	4
Ease of understanding	2	5	3	4
Average (SD)	4.2 (1.30)	4.8 (0.45)	3.6 (0.55)	4.6 (0.55)

**Table 3:** Question 3 ratings.

**Question 3**	**I am taking a lot of different medications. Should I be worried about the number of medications I am on impacting my thinking? If so, which are the most harmful?**			
	**Clinician 1**	**Clinician 2**	**Clinician 3**	**AI**
Clarity	3	5	4	5
Accuracy	4	5	5	5
Relevance	4	5	5	5
Depth	2	3	5	5
Ease of understanding	3	3	2	4
Average (SD)	3.2 (0.84)	4.2 (1.10)	4.2 (1.30)	4.8 (0.45)

**Table 4:** Question 4 ratings.

**Question 4**	**How can I help my loved one with dementia stay living at home as long as possible?**			
	**Clinician 1**	**Clinician 2**	**Clinician 3**	**AI**
Clarity	4	5	4	5
Accuracy	4	5	4	4
Relevance	4	5	4	5
Depth	4	4	4	4
Ease of understanding	3	5	4	4
Average (SD)	3.8 (0.45)	4.8 (0.45)	4 (0)	4.4 (0.55)

**Table 5:** Question 5 ratings.

**Question 5**	**What are the best resources for caregivers of people with dementia?**			
	**Clinician 1**	**Clinician 2**	**Clinician 3**	**AI**
Clarity	4	4	5	5
Accuracy	4	4	5	5
Relevance	4	5	5	5
Depth	3	3	4	4
Ease of understanding	4	4	4	5
Average (SD)	3.8 (0.45)	4 (0.71)	4.6 (0.55)	4.8 (0.45)

**Table 6:** Averages and standard deviations per response type.

	**Clinician 1**	**Clinician 2**	**Clinician 3**	**AI**
Clarity	4.0 (0.71)	4.8 (0.45)	4.4 (0.54)	4.8 (0.45)
Accuracy	4.4 (0.55)	4.6 (0.55)	4.6 (0.55)	4.6 (0.55)
Relevance	4.4 (0.55)	4.8 (0.45)	4.6 (0.55)	4.8 (0.45)
Depth	3.6 (1.14)	3.2 (0.84)	3.6 (1.14)	4.2 (0.45)
Ease of understanding	3.0 (0.70)	4.2 (0.84)	3.6 (1.14)	4.4 (0.55)
Total average (SD)	3.9 (0.59)	4.3 (0.67)	4.2 (0.52)	4.6 (0.26)

For the first question, the AI generated response was rated first overall for ease of understanding, second overall for depth, and tied for last for clarity, accuracy, and relevance. Overall, for the first question, the AI generated response was rated third out of four possible answers with a score of 4.2/5 (SD = 0.45). For this question, the rating clinician correctly identified the AI generated response noting that each of the clinicians' responses sounded as if they were written by trained professionals where the AI generated response did not.

For the second question, the AI generated response was tied for first in clarity, accuracy, relevance, and depth and was second in ease of understanding. Overall, the AI generated response was ranked as the second best of four possible responses with an average score of 4.6/5 (SD = 0.55). The rating clinician again correctly identified the AI generated response stating that two of the other responses used a very similar language to other clinicians while the AI and other clinicians' response seemed to contain “internet information” and had too much extraneous content.

For the third question, the AI generated response was tied for first for clarity, accuracy, relevance, and depth and first overall for ease of understanding. Overall, the AI generated response to this question was ranked first out of four responses with an average rating of 4.8/5 (SD = 0.45). The rating clinician did not correctly identify the AI generated response for this question. They noted that one of the other responses began with the word “yes” which they did not expect from clinicians.

For the fourth question, the AI generated response tied for first in clarity, relevance, and depth, tied for second for ease of understanding, and tied for last for accuracy. Overall, the AI generated response achieved a total score of 4.4/5 (SD = 0.55), which placed second out of the four total responses. The rating clinician correctly identified the AI generated response to this question noting that information contained in the AI's response seemed “a little bit more general” than the other responses.

For the fifth question, the AI generated response was rated highest overall for ease of understanding, and tied for first overall for clarity, accuracy, relevance, and depth of the response. Overall, the response was rated as the best of the four responses, achieving an average score of 4.8/5 (SD = 0.45). The rating clinician again correctly identified the AI generated response, stating that the information contained in the answer was good but focused more on online resources that could be difficult for patients to access.

Cumulatively, AI generated responses were rated as the highest overall for ease of understanding and depth across all responses. The AI generated responses were tied for first for clarity, accuracy, and relevance. The overall rating for the AI generated responses was 4.6/5 (SD = 0.26) whereas the clinicians' responses were rated as 4.3 (SD = 0.67), 4.2 (SD = 0.52), and 3.9 (SD = 0.59) out of 5, respectively. Furthermore, for each question individually, and all questions cumulatively, AI responses had lower standard deviation values than those of the clinicians, suggesting greater consistency in the AI generated response ratings. The standard deviation of AI responses was also tied for the lowest overall for clarity, accuracy and relevance and was the lowest overall for depth and ease of understanding; AI responses were tied for the best in terms of consistency related to all five subcategories. Finally, the rating clinician correctly identified the AI generated answers 80% of the time.

## Discussion

Overall, generative AI's responses to commonly received patient questions had the highest overall average ratings of all responses and placed first overall in terms of depth and ease of understanding, and tied for first in clarity, accuracy, and relevance. AI responses were rated highest on two of the five questions and placed no worse than third overall. AI answers had lower standard deviations for each of the five questions individually and overall, suggesting that its responses were more highly rated and consistent than those produced by clinicians. Furthermore, AI responses were most consistent with regard to depth and ease of understanding and tied for the most consistent for their clarity, accuracy, and relevance. In summation, AI responses were generally rated more highly and consistently on each question individually and overall than clinician answers to hypothetical patient questions.

Although AI was found capable of producing good responses to potential patient questions, a rater was correctly able to identify the AI generated response for four out of five questions suggesting that these answers were easily distinguishable from those of actual providers by an appropriately trained clinician. Per the rater's responses, AI responses appeared identifiable because (1) they did not use language typical of clinicians and (2) contained superfluous information or data that appeared computer generated. Although the rater was mostly able to discern AI from clinician created responses, they noted that AI and clinician responses were not easily distinguishable on multiple occasions.

### Potential benefits of AI

If utilized correctly, AI has the potential to significantly impact patient healthcare. First, as demonstrated in this study, generative AI can quickly and accurately answer complex questions and respond to various healthcare scenarios in an increasingly human like manner. When given adequate prompts and instructions, generative AI can rapidly analyze and summarize large amounts of data and can even be trained to respond to questions with simplified, patient friendly language. AI's ability to rapidly synthesize and generate large amounts of information has the potential to significantly improve both patient and clinician experiences. From a patient perspective, AI technologies may help patients and their families gain insight into various medical and mental health conditions by analyzing and summarizing the most relevant information on a given topic quickly and accurately. Additionally, when provided ample information, AI has demonstrated capabilities to assist with creation of basic treatment plans for certain medical and mental health disorders (Wang et al., 2019; Kneepkens et al., 2022; Peretz et al., 2023). Clinically, the speed, breadth and accuracy of information provided by AI can help providers increase their clinical knowledge, create routine healthcare documentation, generate clinical reports, or formulate basic treatment plans. With assistance from AI, it may be possible for trained clinicians to serve more patients while simultaneously providing each with in-depth, highly personalized care.

Although there are legitimate concerns about the overall accuracy of AI, software based on language learning models continues to evolve and improve. As AI technologies are trained to respond to increasingly diverse and complex questions and scenarios, their accuracy will inevitably improve. Specifically, if experts from various healthcare fields create prompts from which to train AI and correct information generated by it, the technologies will become more accurate, produce more clinically relevant data, and respond in a more human like way. So, despite current reservations about the accuracy of AI technologies, the language learning models on which many AI models are based will allow for continued evolution so that they may eventually be trained to produce increasingly accurate and clinically relevant information.

Finally, if properly leveraged, AI technologies have the potential to increase healthcare literacy, especially in those of lower socioeconomic status. Research has repeatedly shown that those of lower SES are more likely to have poorer health, engage in less healthy behaviors, and have increased medical utilization rates compared with those of higher SES. Theories investigating the relationship between SES and healthcare literacy have shown that the abilities to adequately appraise healthcare information and navigate the healthcare system are lower amongst those of lower SES. AI potentially could close this healthcare literacy gap by providing individuals of lower SES free, comprehensive, and easily accessible healthcare information and resources. Although some AI technologies do require payment to use their services, many currently offer free computer and mobile software. With AI's ever improving abilities to review and synthesize complex medical and mental health topics into easily understandable language and even create basic treatment plans for certain disorders, it has the potential to provide patients increased access and insight into complex medical information and utilize personalized treatment plans. Additionally, if prompted properly, AI may also be able to assist patients with navigating healthcare systems (i.e., understanding medical referral processes, managing medical insurance, etc.), something which can be especially complicated for older or cognitively impaired patients. Being free and easily available, AI can provide patients with information on a wide range of healthcare topics that are otherwise inaccessible, which may help increase overall equity in healthcare.

### Drawbacks of AI

Some notable concerns about current AI technologies include potential difficulties in differentiating AI from human generated speech and the seductive nature of these technologies. Overall, concerns have been raised about people's abilities to separate AI from human generated speech. In fact, a recent study of 529 respondents who listened to AI and human generated speeches (Mai et al., 2023), found that listeners were only able to detect the AI generated speech 73% of the time, meaning that over 1/4 of respondents were unable to differentiate between human and AI generated speech. While respondents trained to recognize AI-generated speech fared slightly better (3.8% improvement) at distinguishing AI from human speech, the improvements were modest, and no other interventions proved impactful. Results from the current study reinforce the potential difficulties of decerning AI from human speech, as even a trained clinician occasionally struggled to separate AI responses from answers provided by trained professionals. Practically, difficulties differentiating AI and human generated speech are concerning as they make it increasingly likely that people mistake AI generated information for that of trained professionals, which could result in the proliferation of inaccurate, incomplete, or harmful information. These concerns about susceptibility to believing information from AI generated text are especially salient for older adults who, partially attributable to diminished cognitive functioning/decision capacity, poorer physiological wellbeing, and lower literacy, may be especially vulnerable to confusing AI for human generated speech (James et al., 2014). Such inappropriate use of AI without oversight from trained clinicians may inform unsafe decision-making based upon misinformation and reduce efficiency/efficacy of clinical interactions as any misconceptions or misinformed patient decisions are addressed.

Additionally, AI technologies can be seductive, leading people to trust information produced by these systems more than they should. Although, in this study, AI generated responses were generally rated as clear and accurate, research has shown that generative AI technologies can be susceptible to providing inaccurate, misleading, and biased information (Augenstein et al., 2023; Wu et al., 2023; Berşe et al., 2024). For instance, an analysis of 49 studies on unsupervised errors made by ML technologies found that 22 of the 49 studies contained demonstrable errors in prediction classification made by AI (Shepherd and Majchrzak, 2022). Further, because language learning model-based AI systems are trained on data created by humans, studies have found that AI is susceptible to similar errors in reasoning as actual people (Hagendorff et al., 2022). Perhaps most concerningly, due to the lack of available data on certain patient populations with which to train AI, information created by AI has shown a potential for bias and discrimination (Ferrer et al., 2021). Despite these notable pitfalls, research has consistently shown that, due to a combination of perceived efficiency, expertise, and impartiality, people tend to trust technologies, including AI, to provide them accurate information.

Limitations in technological access and literacy may limit the usability of AI technologies with certain patients. Research has shown that technology literacy, is poor amongst older adults and individuals of lower socioeconomic status which can impact people's acceptance and adoption of emerging technologies in their daily life and healthcare (Choi and DiNitto, 2013; Watkins and Xie, 2014; Lee et al., 2022). Individuals with diminished technological literacy have greater difficulty accessing/using technology, report less positive attitudes toward emerging technologies, and may even avoid using them in healthcare settings (Ardito and Rabellino, 2011; Chipidza et al., 2015). For instance, a study of lower income disabled/homebound adults and older adults use of Internet based health care services found that they were significantly less likely than the general US population to use these resources due to: (1) lack of exposure or access to computer/internet technologies (2) limited financial resources and (3) having medical, psychological, or cognitive difficulties that limited their ability to use these resources effectively. Related to the last point, using generative AI technologies effectively requires basic knowledge of prompt creation to illicit the best and most accurate information; due to a combination of poor technological literacy and physical/cognitive health difficulties people may struggle to create the prompts to use AI effectively.

The lack of human interaction with AI technologies may also limit their utility. Studies have found that a positive doctor-patient relationship is linked to greater treatment compliance and improved healthcare outcomes (Murdoch, 2021). A strong alliance between patients and providers is associated with improved and lasting treatment outcomes, regardless of the type or length of the intervention used (Murdoch, 2021). The importance of doctor-patient relationship and therapeutic alliance to medical and mental health treatment outcomes raises questions about the potential negative impact of increased AI involvement in healthcare. Specifically, with limited human interaction, will generative AI technologies ultimately have negative impacts on patient engagement and satisfaction with their healthcare services? Additionally, will the lack of interpersonal connection between patient and provider lead to reduced treatment compliance? Given these salient concerns, additional research is needed on the potential impact of AI technologies on relationships between patients and providers and their ultimate effect on healthcare outcomes.

Finally, because AI technologies are trained with mass amounts of real-world patient data, there are concerns related to privacy and data security (Murdoch, 2021). Although many of the AI technologies used in the medical fields are the product of academic research environments, commercialization of AI and the involvement of private entities is becoming increasingly common. Whereas, academic research institutions are usually beholden to set ethical and legal standards regarding data management, including the protection of patient health information, procedures for managing and maintaining private patient healthcare information in the private sector can vary and often involve less institutional oversight and control. Further, previous studies on AI use in the private sector have found that patients were not always afforded agency over use of their healthcare information in research, including certain instances where their information was used without overt consent (Murdoch, 2021). Additionally, there are differing regulations about the location and ownership of servers used by private corporations to store patient health information, leading to increased concerns related to privacy, confidentiality, and patients' rights to access their own information. Still in its infancy, significant concerns remain about the use of AI and the protection of patients' privacy and confidentiality in healthcare settings.

### Potential solutions to AI pitfalls

A potential solution to some of the problems posed by increased involvement of AI in patient treatment may be ameliorated by trained clinicians taking a more active role in the development and use of AI technologies in healthcare. By increasing their involvement in the development of AI technologies, trained clinicians can ensure that data produced by generative AI is comprehensive, accurate, and safe. Specifically, because generative AI technologies require large amounts of patient data from which to learn, clinical researchers can ensure that data provided to the systems is both accurate and relevant with regards to the most updated healthcare knowledge and treatment standards. For instance, a study involving human-in-the-loop development (i.e., training of AI with a multidisciplinary team of providers) of AI for use in healthcare for adolescents with idiopathic scoliosis has served as a proof of concept (Shi et al., 2023). This study illustrated that expert-trained large language models facilitated shared decision-making about treatment between patients and providers. The inclusion of expert-input on the training of AI allows for a safer, more effective, and better informed use of AI in healthcare. Domain-specific expert vetting is thus essential for the ethical implementation of AI in clinical settings. Additionally, researchers trained in management and upkeep of databases involving patient health information can also help ensure that patient privacy and confidentiality are maintained while continuing to develop these AI systems. Finally, providers working directly with patients in clinical settings may be able to integrate salient information about patients' healthcare histories and current presenting symptoms, diagnoses, and/or presenting complaints to assist with increasing diagnostic accuracy and creating personalized and expedient treatment plans using AI.

### Limitations and future directions

A limitation of the current study is that only a few questions were used to generate AI and clinician responses. It is possible that using a greater number of questions spanning a wider range of topics and scenarios will allow for a more accurate assessment of generative AI's capabilities compared to those of trained clinicians. Relatedly, the current study largely made use of fact based, close-ended questions, which given AI's capabilities of digesting and synthesizing large amounts of information, may not have been challenging enough to test the software's true capabilities. Future research would benefit from examining AI's capabilities in analyzing and correctly responding to complex and multifaceted neuropsychological questions and patient profiles. Next, this study only included three clinician respondents and one rater. Future studies would benefit from more clinicians generating and rating responses. Due to the limited number of clinician respondents and raters, this study was only qualitative in nature; future studies would greatly benefit from increased data in order to provide true quantitative information on the capabilities of AI technologies vs. trained clinicians. Further, it would be useful to examine the perceived quality of AI vs. clinician responses from the perspective of patients and their families and see how their responses compared to trained clinician raters. Finally, in this study, length parameters were created for AI responses to reduce the likelihood of identification; future studies could benefit from examining responses of AI that are unconstrained by word counts to assess its true capabilities.

Future directions include the following:

- Train the AI model to produce better responses to patient questions by ensuring it is utilizing the most updated and accurate scientific data to answer questions. It would also be useful to analyze repeated AI responses to the same questions to see if it produces consistent answers or if it improves with repetition.
- Utilize a greater number of queries, including complex and multifaceted neuropsychological questions, to truly evaluate the capabilities of AI compared to trained clinicians. Additionally, inclusion of a greater number of questions will allow for more rigorous, “gold standard” statistical analysis to be implemented to compare AI and clinician generate responses.
- Include more rating clinicians to evaluate inter-rater concordance for both AI and clinician generated responses. Inclusion of clinicians from different specialty areas would help determine if AI performed differently compared to providers in other fields of study.
- Evaluate and compare the responses of AI and clinicians to common patient queries as rated by older patients and their families; these results should also be compared to clinician ratings of the same questions to determine how their perceptions differ.
- Examine responses of AI that are unconstrained by word counts and evaluate these responses against those of trained clinicians.

## Conclusions

Overall, in this study, AI was shown capable of producing good responses to potential patient questions when compared to those of trained clinicians, with the responses being rated as the best on several occasions. However, AI responses were distinguishable from clinicians in most instances, suggesting that, at least to a trained clinician, AI may still lack a human like quality to its responses. Overall, we believe that this research can serve as a basis for larger studies in which our team and others can begin to more rigorously examine the performance of different AI programs in answering common healthcare related questions. Such studies, in turn, can be used to improve the technology in assisting clinicians to provide improved care to a larger number of patients.

## Data availability statement

The raw data supporting the conclusions of this article will be made available by the authors, without undue reservation.

## Author contributions

IM: Conceptualization, Formal analysis, Methodology, Writing – original draft. CM: Visualization, Writing – original draft. EE: Writing – review & editing. JM: Data curation, Visualization, Writing – review & editing. SM: Data curation, Visualization, Writing – review & editing. SH-H: Data curation, Visualization, Writing – review & editing. MC: Data curation, Visualization, Writing – review & editing. PP: Supervision, Writing – review & editing.
