# Supplementary material for: Doctor AI? A pilot study examining responses of artificial intelligence to common questions asked by geriatric patients
Source: Front Artif Intell. 2024 Jul 25;7:1438012. doi: 10.3389/frai.2024.1438012 (PMC11306168; doi:10.3389/frai.2024.1438012)
Supplement: Supplementary file 1 [file Table_1.docx]

Appendix

| **Table 1** |  |  |
| --- | --- | --- |
| *Which Questions were chosen as AI Generated?* | | |
| Question Number | Response Chosen | Rationale for Selection |
| 1 | AI | "The other responses “sound” like clinician s more. The 4th (*Clinician 4*) response sounds like what I would say to a patient over the phone before scheduling. 2 (*Clinician 1*) and 3 (*Clinician 2*) have the specifics that clinician s typically include. The first one does not mention neuropsychological services so seems unlikely that a clinician would have written it." |
| 2 | AI | "1 (*Clinician 2*) & 2 (*Clinician 1*) have information that I have said almost verbatim, so it seems like a real person said them. I had a hard time determining which of the other two I thought was AI. Both seem to have “internet” information and have extraneous information that isn’t necessary for that particular conversation." |
| 3 | Clinician 1 | "My primary reason for choosing #4 (*Clinician 1)* is that it starts with “yes”. I think clinician s tend to be less direct in their responses to these types of questions." |
| 4 | AI | "This one was difficult because there are so many different ways to answer the question. But the 2^nd^ (*AI response*) answer seemed a little more general perhaps? Whereas the others felt more like what you would write in a report." |
| 5 | AI | "The suggestions in #4 (*AI Response*) are good, but they are focused more on online resources, which may be difficult for dementia caregivers to access, and a person would know that." |

| **Table 2** |
| --- |
| *Questions Used To Score Each Response (1-5 Likert Scale)* |
| Question |
| Rate the clarity of the response? |
| Rate the accuracy of the response? |
| Rate the relevance of the response? |
| Rate the depth/breadth of the response? |
| How easy was the response to understand? |

| **Table 3** | | |  |  |  |
| --- | --- | --- | --- | --- | --- |
| *AI and Clinician responses to study questions* | | |  |  |  |
|  | AI | Clinician 1 | | Clinician 2 | Clinician 3 |
| Question 1 | If you suspect your loved one has dementia, it's important to consult a medical professional for an accurate diagnosis. Start by scheduling an appointment with a primary care physician or a neurologist. They can perform assessments, tests, and evaluations to determine the presence and type of dementia. Early diagnosis allows for better management and planning.  Treatment options vary based on the type and stage of dementia. There's no cure, but medications and interventions can help manage symptoms and slow progression in some cases. Medications like cholinesterase inhibitors and memantine may be prescribed. Non-pharmacological approaches include cognitive stimulation, physical activity, and maintaining a healthy diet. Creating a safe and supportive environment at home is also crucial.  Support groups and counseling can provide emotional assistance for both caregivers and individuals with dementia. Legal and financial matters should also be addressed early on. Remember, each person's situation is unique, so consult with healthcare professionals to determine the best course of action. | Next steps would include meeting with your primary care doctor or neurologist for a medical evaluation first to appreciate if there are medical factors that may be causing changes in memory.  The medical provider may also perform a cognitive screening to consider if there has been a possible reduction in cognitive abilities that might objectively corroborate the idea that your loved one is performing worse than would be expected given his/her age.  Your primary care doctor or neurologist may also refer you to a neuropsychologist or Geropsychologist for a more in-depth evaluation to assist with diagnosis and disposition/treatment planning.  Treatment considerations depend on what may be the cause of the dementia, if your loved one is in fact diagnosed as such.  Treatment may include dietary supplementation (B and D vitamins, iron; protein, calories), exercise as tolerated, reduction or cessation of unhelpful health habits (smoking, not wearing CPAP for OSA, not using recreational drugs, alcohol, sugary soft drinks and snacks), reducing fall risk (using walker, taking BP medication, staying hydrated, pause before immediately walking after standing from seated position, not having small pets to trip over), possible additional diagnostic work ups (genetic testing, spinal tap, specialized imaging for amyloid or tau), cognitive rehabilitation/memory training (if early dementia), and medications (acetyl cholinesterase inhibitor, glutamate antagonist, or monoclonal antibodies). | | A) Complete labs for metabolic functioning (e.g., vitamins B12 and D, thymine, thyroid, A1C, liver and kidney functioning, etc.) and undergo a medication review, to rule out reversible causes of cognitive difficulties.  B) Undergo a neuropsychological evaluation to determine etiology and neurocognitive strengths and weaknesses.  C) Increase level of supervision and support. Individuals with dementia often do better with routines and in familiar, structured environments. Outside the context of well-learned routine, the patient is likely to have difficulty in unfamiliar environments and/or when confronted with a novel challenge. Therefore, they might be at an increased risk for encountering/creating dangerous situations, such as mishandling electrical appliances, leaving doors open or unlocked, and so forth.  D) Depending on etiology, the addition of a medication aimed at slowing the progression of dementia symptoms, such as a cholinesterase inhibitor and/or NMDA receptor agonist may be of benefit, unless medically contraindicated.  E) Ensuring that the patient and/or their family are connected to Social Work to assist them in completing an Advanced Directive/future planning paperwork, linking them with services (i.e., respite, home health, community resources), and providing psychoeducation about caregiver support and burnout. | The first step is to discuss your concerns with your loved one’s PCP who can order laboratory work to rule out possible reversible causes of dementia, as well as refer your loved one for additional workup which may include consultation with a geriatrician, neurology, neuropsychology, and neuroradiology. If the PCP has not done so already, a cognitive screener may be completed prior to making referrals. Treatment options will be discussed with you and your loved one based upon findings from the comprehensive workup. |
| Question 2 | Prevagen claims to improve memory and cognitive function by containing a protein found in jellyfish. However, scientific evidence supporting its effectiveness is limited and inconclusive. Clinical studies haven't consistently shown significant memory improvements. Considering the lack of robust evidence and the cost, it might not be worth the expense. It's advisable to consult a healthcare professional before trying any supplements for memory enhancement. | As of 2021, I am aware of 1 clinical study of Prevagen supporting its efficacy. The study has a number of limitations. Thus, this possibly pharmacologically active therapeutic is at best understudied clinically and at worst makes claims likely exceeding what its clever direct to consumer marketing has suggested. I cannot recommend it and do not believe it is worth the cost. | | A) Prevagen has not been shown to improve memory beyond that of a placebo. Thus, I would say the cost is not worth it. If you strongly believe that it is helping you, I suggest that you inform your prescribing providers that you are taking it to ensure there are no interactions with other medications you are on. | It is unlikely that Prevagen, which is considered a supplement, will improve your memory. There are medications approved by the FDA for the treatment and management of dementia symptoms. However, these medications are aimed at slowing the disease process and generally do not lead to improvements in memory. |
| Question 3 | Taking multiple medications, known as polypharmacy, can potentially impact cognitive function, especially in older adults. Medication interactions, side effects, and individual sensitivities can contribute. Medications with strong anticholinergic effects (e.g., some antihistamines, antipsychotics) are more likely to affect thinking. It's essential to consult a healthcare professional to review your medication regimen, identify potential cognitive effects, and discuss possible adjustments to minimize risks while effectively managing your health conditions. | Yes. Clinically, this is called polypharmacy and there is a risk that taking too much and or certain kinds of drugs can definitely impact your thinking skills. Those with strong anti-cholinergic impacts, such as antihistamines, tricyclic antidepressants, anti-emetics, muscle relaxers, medicines used for urinary incontinence, neuroleptics, and medicines used to treat Parkinson’s disease. | | A) There are some medications that can impact cognition/thinking skills. Some of the most impactful include benzodiazepines, anticholinergic medications, and opiates. You should undergo a medication evaluation with a pharmacist. This can help determine if you are taking any benzodiazepines, or other medications that can impact cognition. | It is always important to be mindful of possible medication interactions and polypharmacy and to have ongoing discussions with your prescribing physicians as well as your pharmacist about these concerns. Not only can these periodic reviews be beneficial for reducing possible medication interactions and the effects of polypharmacy, but they can also be opportunities to discuss simplifying your medication regimen. There are several medications which have cholinergic effects and should be monitored and if at all possible, avoided in the elderly, due to their adverse effects on cognition. |
| Question 4 | To help your loved one with dementia remain at home, create a supportive environment. Simplify the living space to reduce confusion, use labels for important items, and establish routines. Enhance safety by removing hazards and installing locks or alarms. Provide memory aids like calendars and whiteboards. Hire caregivers or home health aides to assist with daily tasks. Consider adult day programs to provide social engagement and respite for caregivers. Maintain their physical and mental health through regular exercise, a balanced diet, and cognitive activities. Utilize technology like medication reminders and GPS trackers. As their needs change, be prepared to adapt the care plan and seek professional advice. Regular communication with doctors and support groups can provide guidance and emotional assistance. | Regular visits with medical team to manage medical factors and consider therapeutic options to support brain function and general health, consider home health if/when a decline in ability for self-care warrants it, work to support a healthy diet and exercise plan, work to reduce engagement in or work towards cessation of unhealthy behaviors such as alcohol and smoking, look around and reduce risk for falls wherein possible (see above; Q#1), engage the loved one in regular social activity and opportunities for him/her to engage critical thinking, work to connect loved one with one or more purposes that they find meaningful and rewarding, consider adult day care /respite day care on occasion, consider engaging in caregiver support groups, encourage caregivers to learn about dementia and care-giving such as educational offerings thru the Alzheimer’s Association or reputable books such as the 36 hour day. | | A) Increase level of supervision and support. Have someone with them to provide assistance throughout the day.  B) Provide structure via daily routines.  C) Avoid making big changes to the living environment and capitalize on the patient’s familiarity with their living environment.  D) Caregiver support to prevent burnout.  E) Home health may be able to assist with some ADLs. | Ensuring that your loved one has adequate supports to safely live independently is of the utmost importance. Addressing physical, cognitive, and behavioral limitations is key. Physical limitations are important to consider to reduce the risk of physical injury. Your loved one may need assistive devices to maximize their ability to safely perform basic and instrumental activities of daily living. Additionally, equipping the home with safety accommodations like a shower chair or grab bar in the shower or tub can help increase your loved one’s safety when bathing independently. Cognitive limitations are also important to consider for your loved one’s safety. For example, if forgetting to take their medication is something that your loved one struggles with, then supports and techniques to help your loved one better manage their medication should be identified. This can range from simple reminders or the use of a pillbox with reminders to the need for home health services where a nurse may come to your loved one’s home and directly assist with this task. Finally, it is important to address any behavioral limitations that your loved one may present with that may become a safety risk. Some examples include sundowning which may involve increased confusion and agitation as the day progresses. This can be very stressful and even frightening for some patients with dementia. Another example of a behavioral safety concern is wandering behavior, which potentially places your loved one at risk for getting lost, injured, or vulnerable to external threats. Home health may also be an option in these cases where there is a designated aide who will help redirect your loved from wandering. |
| Question 5 | Caregivers of people with dementia can find valuable resources through organizations like the Alzheimer's Association and AARP. Online platforms like Caregiver Action Network and Family Caregiver Alliance offer information, support groups, and educational materials. Local senior centers, community health services, and hospitals often provide workshops and support groups. Healthcare professionals can offer guidance on care strategies. Utilize apps like CaringBridge for coordinating care among family members. Connecting with others who are in similar caregiving roles can offer emotional support and practical advice. | Working with medical team who has experience working with persons with dementia to facilitate connection to reputable resources within and outside of hospital setting, to include local or national Alzheimer’s Association (found by website; with phone number and address), dementia caregiver support groups offered by/housed within hospital, reputable websites such as Alzheimer’s or Stroke Association, and reputable reading materials such as 36 hour day, a dignified life, creating moments of joy, or living your best with early stage Alzheimer’s. | | A) Caregiver support groups and programs via hospitals/VA/organizations. Groups provide a place caregivers can find validation, support, and advice.  B) The 36 Hour Day book, and other reading material via the Alzheimer Association and other groups.  C) Alzheimer Association has a large support network.  D) Connecting the family with Social Work can also provide another avenue for patients and their families to gather additional resources out in the community.  E) Respite services via hospitals/VA/organizations. | The best resources for caregivers of people with dementia fall into several categories. Informational resources are available from treating professionals, professional organizations (e.g., the Alzheimer’s Association), reference books (e.g., The 36-hour Day), and support groups. Caregivers need all of the social support that they can get, whether this includes enlisting the help of other caregivers, or taking advantage of such resources as adult day programs and respite care. While caregiving offers many rewards, it also comes with some challenges. The caregiver’s well-being is very closely tied to the welfare of the affected patient, so it is very important to be aware of and make attempts to mitigate any signs of caregiver burnout. In some cases, caregivers may benefit from psychotherapy to identify adaptive ways of managing their stress. |
